# Safety of Sofosbuvir and Ribavirin Combination Therapy in a Patient Who Developed Anemia due to Ribavirin

**DOI:** 10.1155/2017/8793895

**Published:** 2017-12-10

**Authors:** Hirokazu Suii, Itaru Ozeki, Ryoji Tatsumi, Masakatsu Yamaguchi, Mutsuumi Kimura, Tomohiro Arakawa, Tomoaki Nakajima, Yasuaki Kuwata, Takumi Ohmura, Shuhei Hige, Yoshiyasu Karino, Joji Toyota

**Affiliations:** Department of Hepatology, Sapporo Kosei General Hospital, 8-5 Kita Sanjo Higashi, Chuo-ku, Sapporo, Hokkaido 060-0003, Japan

## Abstract

Interferon (IFN) and ribavirin (RBV) combination therapy was previously the standard of care for treatment of hepatitis C virus (HCV) genotype 2 infection. But, it often induced hemolytic anemia. In 2014, sofosbuvir (SOF) was approved for the treatment of chronic HCV genotype 2 in Japan. SOF/RBV therapy is more effective against genotype 2 than IFN/RBV therapy. We report a case of a 74-year-old woman with chronic HCV genotype 2b infection. She received five treatments including RBV and IFN therapy before SOF was approved and all of them were ineffective. Therapies that included RBV induced severe anemia and led to discontinuation of treatment. With pegylated IFN/RBV therapy, the maximum change in hemoglobin (Hb) from baseline was −3.7 g/dL. However, SOF/RBV therapy was effective and she achieved sustained virologic response (SVR) with a maximum change in Hb from baseline of only −1.2 g/dL. We also found reticulocyte count was very low during treatment in this case and speculate it was one of the reasons that she developed hemolytic anemia with RBV. In conclusion, SOF/RBV therapy is effective and allowed the patient to achieve SVR. An SOF/RBV regimen is safe and effective for patients who have or are at risk of anemia induced by RBV.

## 1. Introduction

Chronic hepatitis C virus (HCV) infection is a cause of chronic liver disease and hepatocellular carcinoma [1, 2]. Recently, most patients with HCV infection are treated with interferon-free direct-acting antiviral agents (DAAs). Previously, ribavirin (RBV) was commonly used in combination with pegylated interferon (Peg-IFN) for treatment of HCV infection; however it often induced severe hemolytic anemia and patients tended to discontinue treatment even with a reduction of RBV dose. It has been reported that clinical risk factors for severe RBV-induced anemia include impaired renal function, older age, high dose per body weight of RBV, and female sex [3]. Here, we report a case of HCV genotype 2 infection in a patient who previously had severe anemia with IFN/RBV combination therapy. Despite a low reticulocyte count, she was able to achieve sustained virologic response (SVR) with the combination of sofosbuvir (SOF) and RBV.

## 2. Case Report

**Figure 1:**
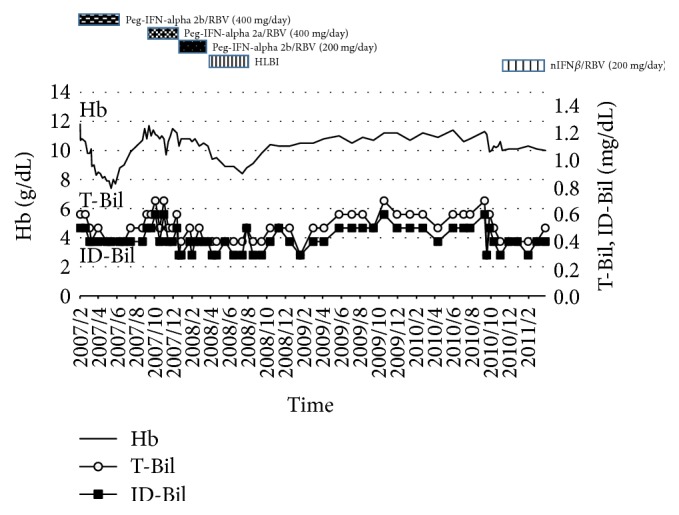
Clinical course of patient (Hb, T-Bil, and ID-Bil). T-Bil: total bilirubin; ID-Bil: indirect bilirubin.

**Figure 2:**
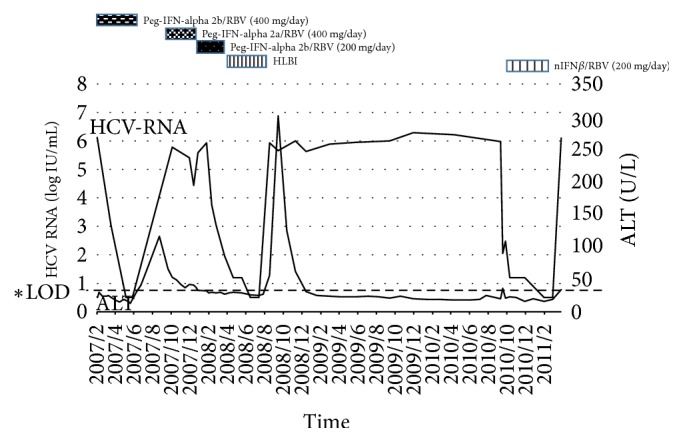
Clinical course of patient (ALT and HCV RNA). ^*∗*^Limit of detection (LOD).

We present a case of a 74-year-old woman. She had a history of surgery to resect a pelvic tumor 30 years ago, which caused massive bleeding and required blood transfusion. One month after surgery, she was hospitalized for 10 months owing to posttransfusion hepatitis. She was subsequently diagnosed with HCV infection. In December of 2006, she visited our hospital for treatment of chronic hepatitis C. Laboratory examination showed her HCV genotype was 2b and pretreatment viral load was 1500 KIU/mL (6.17 log⁡IU/mL). Her* IL28*B genotype showed a single-nucleotide polymorphism (SNP) TT (rs8099917) as major homozygous and the* ITPA* genotype showed SNP CA (rs7270101) as minor allele heterozygous. The clinical treatment progress is as follows (Figures 1 and 2). Initial treatment with Peg-IFN-alpha 2b 80 *μ*g/week and RBV 400 mg/day was started in our hospital in March of 2007. After 10 weeks of therapy with this regimen, she developed severe hemolytic anemia. Her hemoglobin (Hb) level decreased from 12.0 to 8.1 g/dL, requiring discontinuation of treatment.

**Figure 3:**
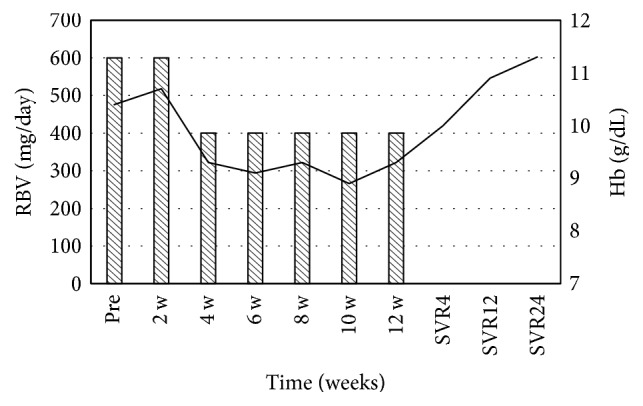
The variation of hemoglobin value with SOF and RBV combination therapy. Sofosbuvir dose was 400 mg/day.

**Figure 4:**
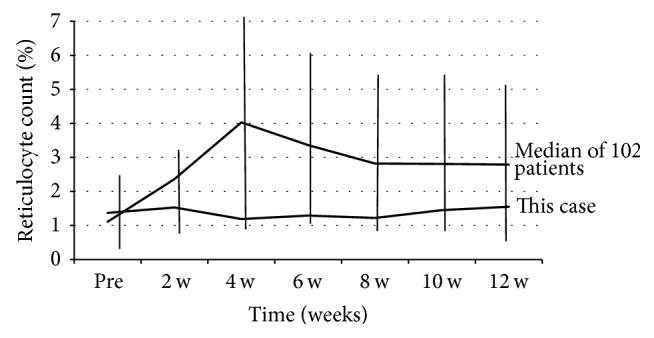
The variation of reticulocyte count during treatment with IFN and RBV combination therapy in this case and median of another 102 patients treated by our hospital.

Secondly, she received Peg-IFN-alpha 2a 80 *μ*g/week with RBV (400 mg/day), but the treatment was not effective and resulted in virological breakthrough. Treatment with the same dose of Peg-IFN-alpha 2b and a 50% dose reduction of RBV (200 mg/day) was then started for 12 weeks (since November 2007). Due to the low dose of RBV, the viral load did not change and SVR could not be achieved. Fourthly, the administration of long-term low dose human lymphoblastoid interferon (HLBI) 6 MU/day was started (since February 2008). After 20 weeks of therapy, a treatment-induced stomatitis developed and this treatment was discontinued. After the fourth treatment, she did not receive antiviral treatment for two years. In October 2010, treatment with nIFN*β* 6 MU/day and RBV 200 mg/day was started. The hemoglobin level was not reduced too much because of the low dose of RBV. She achieved an undetectable HCV RNA status with this treatment, but HCV RNA relapsed 4 months after the completion of treatment. In 2014, SOF was approved for the treatment of genotype 2 chronic hepatitis C in Japan. Starting in September 2015, she was treated with SOF 400 mg/day and RBV 600 mg/day for 12 weeks. This RBV initial dose was the highest dose of her previous treatments. Laboratory results at the start of SOF/RBV combination therapy are shown in Table 1. Since her Hb level decreased gradually, the RBV dose was reduced from 600 to 400 mg/day at 4 weeks (Figure 3). And, in spite of anemia, the reticulocyte count did not respond to the stimuli; it was very low during this treatment in this case (Figure 4).

Although SOF/RBV combination therapy tended to induce hemolytic anemia like previous therapies, she completed 12 weeks of SOF/RBV therapy and finally achieved SVR.

Written informed consent was obtained from the patient for publication of this case report.

## 3. Discussion

IFN and RBV combination therapy was previously the standard of care for treatment of genotype 2 HCV infection [4]. IFN therapy is more effective against genotype 2 than genotype 1 [5, 6]. Approximately 80% of genotype 2 patients who received 24-week Peg-IFN/RBV combination therapy achieved SVR [6–8]. However, RBV often induces severe hemolytic anemia, and IFN can induce side effects such as granulocytopenia and thrombocytopenia and neuropsychiatric effects like depression [9]. These adverse effects are the reason why most patients discontinue treatment of HCV infection with IFN/RBV combination therapy; lack of treatment results in the inability to achieve an SVR. Many patients waited until DAAs were approved. In this case, the patient discontinued multiple previous therapies because of severe hemolytic anemia with RBV. As an older, female patient, the patient did have risk factors for severe RBV-induced anemia. However, her CA* ITPA* genotype (minor allele heterozygous) is less likely to be affected by hemolytic anemia than the CC* ITPA* genotype (major homozygous) [10]. She also had normal renal function. In spite of generally favorable treatment conditions, she could not achieve SVR. The approval of SOF in Japan allowed the patient to achieve SVR with SOF/RBV combination therapy. In a Japanese phase 3 trial, 97% of genotype 2 patients achieved SVR12 and experienced a mean change in Hb from baseline to week 12 of treatment of only −1.7 g/dL [11]. In this case, the maximum change in Hb from baseline was only −1.2 g/dL (Figure 3) whereas, with previous IFN/RBV combination therapy, the maximum Hb decrease from baseline was −3.7 g/dL.

In addition, it is notable that the reticulocyte count was very low during treatment with SOF/RBV in this case (Figure 4). It has been reported that reticulocyte count tends to increase from baseline within the first three weeks in response to anemia after RBV therapy-induced hemolytic anemia [12]. In our hospital, the median variation of reticulocyte count in 102 patients who received SOF/RBV combination therapy was 4.03% at four weeks. All 102 patients had an increase of reticulocyte counts during the first four weeks of therapy. However, this case did not show an increase of reticulocyte count during treatment. This is possibly due to prior bone marrow suppression. This mechanism is not well known. From this case, we can conclude that there is a value in using SOF/RBV combination therapy for the failed cases of therapies including IFN and RBV. Of course, for cases where the hemoglobin value does not rise above 12 g/dL, other treatment options should be considered.

In summary, SOF/RBV combination therapy is an appropriate and effective treatment for patients with chronic HCV genotype 2 infection, especially patients who could not achieve SVR due to severe hemolytic anemia or other side effects caused by RBV and IFN.

## Figures and Tables

**Table 1:** Laboratory results at the start of SOF/RBV combination therapy (2015/9).

*CBC*		
WBC	3.4	×10^3^/*μ*L
RBC	3.3	×10^6^/*μ*L
Hb	10.4	g/dL
Hct	29.9	%
MCV	90.6	fL
Plt	159	×10^3^/*μ*L
Reticulocyte	1.37	%
*Coagulation*		
PT	81	%

*Tumor marker*		
AFP	3.6	ng/mL
PIVKA-II	18	mAU/mL

*HCV viral marker*		
HCV RNA	6.14	log⁡IU/mL
HCV genotype	2b	
*Biochemistry*		
TP	7.2	g/dL
Alb	4.2	g/dL
Ferritin	98	ng/ml
T-Bil	0.9	mg/dL
D-Bil	0.1	U/L
ID-Bil	0.8	U/L
AST	37	U/L
ALT	26	U/L
LDH	227	U/L
ALP	134	U/L
*γ*-GTP	13	U/L
BUN	10.9	mg/dL
Cr	0.63	mg/dL
eGFR	69.1	ml/min/1.73 m^2^
T-chol	199	mg/dL

*IL-28B genotype*	TT	
(rs8099917)		
*ITPA*	C/A	
(rs7270101)		

FIB-4 index	3.38	
Fibro scan^*∗*^	4.3	Kpa (F0 level)
CAP^*∗∗*^	206	dB/m (S0 level)

*Height*	148	cm
*Weight*	49.8	kg

^*∗*^Transient elastography (Fibroscan®). ^*∗∗*^CAP: controlled attenuation parameter.
